# The Missing Part of Seed Dispersal Networks: Structure and Robustness of Bat-Fruit Interactions

**DOI:** 10.1371/journal.pone.0017395

**Published:** 2011-02-28

**Authors:** Marco Aurelio Ribeiro Mello, Flávia Maria Darcie Marquitti, Paulo Roberto Guimarães, Elisabeth Klara Viktoria Kalko, Pedro Jordano, Marcus Aloizio Martinez de Aguiar

**Affiliations:** 1 Institute of Experimental Ecology, University of Ulm, Ulm, Germany; 2 Programa de Pós-graduação em Ecologia, Universidade Estadual de Campinas, Campinas, Brazil; 3 Departamento de Ecologia, Universidade de São Paulo, São Paulo, Brazil; 4 Smithsonian Tropical Research Institute, Balboa, Panama; 5 Estación Biológica de Doñana, Consejo Superior de Investigaciones Científicas, Sevilla, Spain; 6 Instituto de Física ‘Gleb Wataghin’, Universidade Estadual de Campinas, Campinas, Brazil; Institut Mediterrani dEstudis Avançats (CSIC/UIB), Spain

## Abstract

Mutualistic networks are crucial to the maintenance of ecosystem services. Unfortunately, what we know about seed dispersal networks is based only on bird-fruit interactions. Therefore, we aimed at filling part of this gap by investigating bat-fruit networks. It is known from population studies that: (i) some bat species depend more on fruits than others, and (ii) that some specialized frugivorous bats prefer particular plant genera. We tested whether those preferences affected the structure and robustness of the whole network and the functional roles of species. Nine bat-fruit datasets from the literature were analyzed and all networks showed lower complementary specialization (*H_2_'* = 0.37±0.10, mean ± SD) and similar nestedness (*NODF* = 0.56±0.12) than pollination networks. All networks were modular (*M* = 0.32±0.07), and had on average four cohesive subgroups (modules) of tightly connected bats and plants. The composition of those modules followed the genus-genus associations observed at population level (*Artibeus-Ficus, Carollia-Piper,* and *Sturnira-Solanum*), although a few of those plant genera were dispersed also by other bats. Bat-fruit networks showed high robustness to simulated cumulative removals of both bats (*R* = 0.55±0.10) and plants (*R* = 0.68±0.09). Primary frugivores interacted with a larger proportion of the plants available and also occupied more central positions; furthermore, their extinction caused larger changes in network structure. We conclude that bat-fruit networks are highly cohesive and robust mutualistic systems, in which redundancy is high within modules, although modules are complementary to each other. Dietary specialization seems to be an important structuring factor that affects the topology, the guild structure and functional roles in bat-fruit networks.

## Introduction

In the tropics, plant-animal mutualisms such as seed dispersal are vital for ecosystem functioning [1]. A huge body of knowledge has been accumulated on the ecology of those interactions at population level [2]. However, as the properties of a complex system cannot be totally predicted based only on the properties of its elements (in this case, population of animals and plants) [3], if we want to address the importance of seed dispersal as an ecosystem service, and to better understand its role in maintaining biodiversity, it is essential to analyze animal-fruit interactions at the community level, i.e. considering all species at a given locality [4]. Among other tools (such as multivariate analysis), network theory is proving extremely helpful in this task, as it provides a theoretical framework and useful analytical methods to assess patterns of interaction among several species of frugivores and fruits [5]. Network theory provides innovative tools that can be used as surrogates for assessing complex ecological concepts. It is important to say, though, that network ecology does not replace traditional community ecology, but rather complements it, because while the former focuses more on the interactions, the latter focuses more on the species. There are some branches of community ecology, mainly guild theory [6], [7], that dealt with interactions for a long time; network ecology brought tools from complexity theory that made it easier to assess those complex systems.

Unfortunately, what we know about seed dispersal networks is based only on bird-fruit interactions [8], although other animal groups also play important roles [9]. Bats represent a key disperser group that has been neglected so far in network studies, although frugivorous bats and birds are jointly responsible for over 80% of the seed rain in Neotropical sites [10]. Furthermore, bat services are in most cases highly complementary to bird services [11]. This gap in our knowledge needs to be closed quickly, as evidence indicates that different animal groups form species subgroups within mutualistic networks of different kinds, including pollination [12] and ant-plant mutualisms [13]. Therefore our knowledge of seed dispersal will be markedly biased until other animal groups are also studied from a network perspective.

Most studies on bat-fruit interactions have been limited to the population level, i.e. local interactions of single bat species with local fruits, mostly including data from only one site. Consequently, little is known about the community structure of bat-fruit interactions, with very few exceptions [14], [15]. To start with, it is known that within the family Phyllostomidae, specialized frugivory (i.e. complete or strong dependence on fruits for a living) seems to have evolved only once in the species-rich lineage comprising the subfamilies Carolliinae, Rinophyllinae, and Stenodermatinae [16]. Bats of the subfamily Glossophaginae may also feed on fruits, but this part of their diet represents a secondary choice after nectar and pollen; bats of the Lonchophyllinae are almost exclusively nectarivores, and very few reliable records exist on fruits in the diet of the animal-eating Phyllostominae bats [17]. This is probably one reason why, despite all dietary diversification among frugivorous phyllostomids [17], all of them have one or more of five main plant genera as the core of their diet: *Cecropia*, *Ficus*, *Piper*, *Solanum,* and *Vismia* [17].

Considering those five main plant genera, a close genus-to-genus relationship exists between them and some primarily frugivorous bat genera. When their preferred fruits are available, bats of the genus *Artibeus* eat mostly fruits of *Ficus* (Moraceae), whereas *Sturnira* bats select primarily *Solanum* (Solanaceae), and *Carollia* bats feed preferably on *Piper* (Piperaceae) [18]. For phyllostomid bats infrequently used plant species do not play a large role in nourishment [17]. What most phyllostomids do is to shift between species of those five main plant genera, depending on their availability at different seasons [18], [19], [20]. Some of them do also change to a nectar or insect diet at times of fruit scarcity [21]. In what concerns essential inorganic nutrients, some phyllostomids obtain this complement from leaves [22] or muddy water [23].

Such clustering tendencies have been observed at the community level in comprehensive dietary studies of bats in Panama [15]. However, it is important to note that there is substantial geographic variation in those interactions. In Mexico, *Artibeus* bats feed mainly on *Cecropia* [14] and not *Ficus*; this difference may be explained largely by resource availability, as bat figs are not always abundant in Neotropical localities. Moreover, this kind of spatial variability has been expected, based on the theory of geographic mosaics of coevolution [24]: most widespread species are under different selective pressures across their geographical range, having different sets of available partners and, eventually, specializing in different species.

Considering the evidence from population-level studies, bats with narrower diets seem to feed on a subset of the plants consumed by bats with broader diets [25]; therefore, high nestedness [sensu 26] was expected in bat-fruit networks. We also expected low complementary specialization [27]; i.e. bats of different species should feed on relatively similar subsets of plants, considering that only five plant genera form the core of bat diets. Moreover, clustering is probably low in bat-fruit networks: in bat-fruit networks there should only be a few interconnected cohesive subgroups of frugivorous species associated with specific subsets of plants, forming guilds [7]. Furthermore, genus-genus associations between bats and plant are likely to play a decisive role in subgroup formation, although this does not exclude the possibility that each plant genus is dispersed by more than one bat genus.

Ultimately, the combination of high nestedness, low complementary specialization, and low clustering should lead to high robustness in bat-fruit networks, as has been suggested for other kinds of mutualistic networks [28]. In other words, bat-fruit networks should be relatively robust as regards removal of species on either side (plants or bats); i.e. when species are cumulatively removed from one side of the network (e.g. plants) most species on the other side (e.g. bats) should still remain. Furthermore, since primarily frugivorous bats depend on fruits for a living, they probably interact with more fruit species in each network and thus occupy more central positions and play more important roles in maintaining network structure. If this is true, we would expect the removal of primary frugivores to result in larger decreases in the system's nestedness.

Thus in the present study we aimed at testing these hypotheses on the structure and robustness of bat-fruit networks, in order to add an important piece to the puzzle of mutualistic networks and associated ecosystem services. As seed dispersal is a crucial service in the disrupted and fragmented landscapes all over the Neotropics [2], a more complete understanding of its network structure and fragility is of great importance.

## Methods

### Datasets

We used nine datasets on the diet of Neotropical frugivorous bats compiled from the literature. Eight datasets were weighted (i.e. contained data on the frequency of the interactions) and came from fecal analysis conducted in Brazil, Costa Rica, and Peru. We also used one long-term dataset with presence/absence information compiled by E. K. V. Kalko and co-workers on Barro Colorado Island (BCI), Panama (for details see reference list in Appendix S1). Overall, we included only datasets in which interactions had been sampled at least for one year, and in which all frugivorous bat species in the study area were considered, and not only a pre-defined subset (for instance, a single genus or species). As most data on plant consumption were obtained from fecal analysis, some species are lacking, mainly fruits with large seeds that are not swallowed but discarded at the site where the bats chewed the fruits [29]. The data set from BCI is more complete as it includes data from observations, roost inspections, and fruits that were carried by the bats into mist nets.

For our analysis we considered all bat species as seed dispersers, even if a few of them may be actually mainly seed predators; actually, so far only bats of the genus *Chiroderma* are known to feed on seeds [30]. We regard the effect of seed predators in this case as negligible since these bats represent only a very small proportion of all frugivorous species in the area, and they also usually disperse at least some seeds (pers. obs. I. Wagner and E. Kalko).

### Network structure

We organized datasets as adjacency matrices of animals and plants, *A*×*P*, with bat species as *A* rows and plant species as *P* columns, to test for the network structure of bat-fruit interactions (Appendix S2). In the weighted datasets, cell values indicate the number of fecal samples of each bat species that contained seeds of each plant species. In the single binary dataset, cell values are only 0 or 1, i.e. absence of presence of interaction between each bat and plant. Graphs were drawn in Pajek 2.02 [31] and in the package bipartite for R [32].

The index *NODF* in the software Aninhado 3.0 [33] was used to measure the degree of nestedness of each network. *NODF* is a much better nestedness metric than the classic metric *T* [34], because it is more fine-tuned to the original concept, as it is based on the nestedness of all pairs of columns and rows in the matrix [26]. In a nested network, species with fewer interactions are connected to a subset of the partners of species with more interactions. *NODF* values were normalized in order to vary from 0 (not nested) to 1 (fully nested). The significance of *NODF* was estimated with a Monte Carlo procedure. First, we generated 1,000 random matrices from the original matrix, using the null model 2 [10] (null model Ce in Aninhado), in which the probability of interaction between a bat and a plant species is proportional to their total number of interactions (i.e. their degree). Second, we defined the P-value as the proportion of random matrices that had a *NODF* value equal or higher than the value obtained for the real matrix. When no random matrices had higher *NODF* than the real matrix, we defined P<0.001.

In this study, we worked with concepts of specialization from ecological theory (theoretical variables) and from network theory (operational variables). In summary, we aimed at testing predictions based on ecological theory with network surrogates. We used concepts that deal with each network as a whole (network level), and concepts that deal with each species (species level). At the network level, we used the operational concept of “complementary specialization” to test for interaction specialization in the community as a whole. This concept does not take into account dietary preferences or coevolutionary associations, as is usual in ecological theory. It only considers the number of interactions established by a species within a network (i.e. its degree) and how those interactions differ among species. We used the *H_2_'* index [35], which varies from 0 (all species interacting with the same partners) to 1 (each species interacts with a particular subset of partners) to assess complementary specialization. This index has the additional advantage of reducing sampling biases, as it considers a species as specialized, only when it interacts very frequently with another species that has a few other partners in the network. The significance of *H_2_'* was estimated with a Monte Carlo procedure. First, we generated 10,000 random matrices using the null model Patefield [35], in which the interaction frequency between two species is proportional to their total sum of interactions. Second, we defined the P-value as the proportion of random matrices that had a *H_2_'*value equal or higher than the value obtained for the real matrix. When no random matrices had higher *NODF* than the real matrix, we defined P<0.001. All analyses on complementary specialization were made in R with the package bipartite.

In order to test whether feeding preferences of particular bat genera for particular fruit genera [17] produced guilds in the community [7], we used as a surrogate of guild the concept of module, assessed with a functional cartography algorithm for modularity [36]. Modularity is a measure of how much the network is structured in cohesive subgroups of vertices (modules), in which the density of interactions is higher within than among subgroups. Modularity was calculated with the index *M* (from 0, no subgroups, to 1, totally separated subgroups) with a simulated annealing algorithm in the software Netcarto (kindly provided by R. Guimerà upon request); its significance was estimated with a Monte Carlo procedure: 100 random matrices were generated with the null model Ce (null model 2 of [10]), in which the probability of interaction between a bat and a plant species is proportional to their total proportion of interactions (i.e. their degree). Second, we defined the P-value as the number of random matrices that had an *M* value equal or higher than the value obtained for the real matrix. When no random matrices had higher *M* than the real matrix, we defined P<0.001. We used the original bipartite networks in this analysis, following other studies on mutualistic networks [e.g. 12], because unipartite projections change the meaning of links from seed dispersal to niche overlap, and we wanted to assess the guild structure of the networks. As the software Netcarto was made for unipartite networks (in which plant-plant and animal-animal connections are allowed), we created a costume-made procedure for this analysis, combining a MatLab code (for generating random matrices) with a Fortran code (for automating the calculation and compilation of *M*-values). We assessed the consistency of the genus-genus associations between bats and plants at network level in the following way. In all networks, bats of the same genus were found together in the same modules. So we counted in how many networks each bat genus was found in a module that contained plants of its supposedly preferred genus. Then we pooled together data from different networks, and built one 2×2 table for each genus: rows contained the count of modules that followed our prediction and the count of modules that did not; columns contained observed and expected values (equal proportions). Differences were assessed with G tests (with Yates correction).

### Level of frugivory

There are many ecological concepts of dietary specialization at the species level, and we decided to work with the concept of ‘level of frugivory’: the dependence on fruits for living, when considering all kinds of food eaten by the animal species. We followed a concept developed for frugivorous birds [37], and based our classification on the consensus that only bats of the family Phyllostomidae feed on fruits in the Neotropics, and that phyllostomids of the subfamilies Carolliinae and Stenodermatinae depend strongly on fruits for living (category “primary”). Some members of the Glossophaginae take fruits as secondary food (“secondary”), whereas other members of this subfamily and of the subfamilies Phyllonycterinae and Phyllostominae seldom feed on fruits (“occasional”).

A comprehensive dataset on bat-fruit interactions across the Neotropics (365 papers and 4,100 records) was used to refine our classification, as it allowed us to see how many records of frugivory each species has. To build this dataset we started from information published in bat-plant databases [38], and complemented it with literature mostly from South America. In total, our database comprises 365 papers and 4,100 records of interactions. We considered only records with taxonomic resolution to the species level for both bats and plants. This way we separated secondary from occasional frugivores in the nectarivorous subfamilies, based on how often each species has been recorded feeding on fruits.

Finally, we tested how the level of frugivory of each species explained its functional role in the network. We used two network surrogates for functional role, which are explained in the next section. The relationships between level of frugivory and proportion of interactions, and between level of frugivory and betweenness centrality, were tested with Kruskal-Wallis tests.

### Functional roles

Each species in a mutualistic network has a different pattern of interaction, and therefore plays a different role in the functioning and maintenance of its community; this is the species' functional role or Eltonian niche [39]. We assessed the functional role of each species in the seed dispersal network with two network surrogates (see details on the calculations in [40], [41]). The first surrogate for functional role was the species' proportion of interactions, i.e. to how many other species it is connected in the network in relation to the total number of possible partners available (i.e. relative or normalized degree - *k_r_*). Second, we assessed each species' betweenness centrality (*bc*), i.e. the proportion of shortest paths (geodesics) that contain the target species in relation to all existing shortest paths between all species pairs in the network. A path between two species in a network is defined as the number of links from one to the other [41]; a species that is included in a high proportion of geodesics has a central position in the network. Proportion of interactions reflects the species' local niche breadth, whereas betweenness centrality reflects how important its niche is in the whole interaction system. Species with a high proportion of interactions compared to other species in the same network are called ‘hubs’, whereas species with a high betweenness centrality are called ‘connectors’.

### Robustness to extinctions

To test for the robustness of bat-fruit networks to species extinctions, we used a network surrogate obtained from a procedure based on cumulative removals of species from the network at random [42]. First, we removed one species from one side of the network (e.g. plants); when another species from the other side (e.g. bats) was connected only to the removed species, it was also removed from the network (i.e. a secondary loss). Then another species was also removed, without putting the first one back, until all species from the chose side were removed. In this way an extinction curve was generated by plotting the number of remaining species on the one side against the cumulative number of species removed from the other side. The same procedure was carried out for both sides of each network, resulting in one curve for plants and another for animals. The area below each curve (*R*) was calculated as a measure of the robustness of the whole system. *R* = 1 corresponds to a very slow decrease in the curve, and thus represents a system in which most plants remain after the removal of most animals, or vice-versa; *R* = 0 corresponds to a very fast decrease in the curve, and thus represents a system that already collapses after the first few species have been removed. We ran 100 randomizations for each network. This analysis was carried out in the package bipartite for R. On the one hand, it is known that some of the studied bat species, for instance, do also feed on other item such as pollen, nectar and insects [20]. On the other hand, some of the studied plant species are dispersed also by other kinds of animals, such as birds or primates [43]. Therefore, it is important to say that species removals in our simulations do not necessarily represent real extinctions in nature, as we were dealing with seed dispersal systems, and not whole ecological communities. Removals in our study represent exclusion of particular species from the seed dispersal service.

At species level, we wanted to test how the removal of particular species affected the whole network structure, depending on its level of frugivory. To do this we also simulated single-species removals in each network in Ataque 1.0 (M.A.M. de Aguiar and F.M.D. Marquitti, designed for this study) using a jackknife procedure. We excluded one species and its interactions in a network at a time, and observed the percentage of change that this removal caused in the degree of nestedness (*NODF_r_*). This value was calculated as: *NODF_r_* =  (*NODF_obs_* – *NODF_ori_*)/*NODF_ori_*, where *NODF_obs_* is the observed value of *NODF* after a species was removed, and *NODF_ori_* is the original value of *NODF* of the complete matrix. The relationship between level of frugivory and *NODF_r_* was assessed with Spearman correlations. As nestedness is hypothesized as improving network robustness [28], we assumed that a decrease in nestedness has a negative effect on the network by decreasing its robustness.

## Results

Total network size (number of bat and plant species) varied from 12 to 69 (average ± SD: 31±18). Corroborating our first prediction, all nine networks were nested (*NODF* = 0.56±0.12, all P<0.01) (Table 1). Furthermore, complementary specialization was also significant (*H_2_'* = 0.37±0.10, all P<0.001).

**Table 1:** Parameters measured in the nine studied bat-fruit networks: species richness, number of plant species, number of bat species, degree of nestedness (*NODF*), complementary specialization in the interactions (*H_2_'*), modularity (*M*), number of modules found (*Modules*), and robustness to the extinction of bats or plants (*R*). *H_2_'* could not be calculated for the network Kalko BCI as it has only binary data.

Network	Richness	Plants	Bats	NODF	H_2_'	M	Modules	R (bats)	R (plants)
Faria 1996	23	15	8	0.55	0.36	0.33	4	0.59	0.69
Garcia et al. 2000	20	14	6	0.41	0.39	0.44	5	0.41	0.58
Gorchov et al. 1995	37	26	11	0.67	0.30	0.24	4	0.69	0.84
Hayashi 1996	19	12	7	0.53	0.51	0.32	4	0.55	0.63
Kalko BCI	69	47	22	0.39	n/a	0.36	6	0.65	0.74
Lopez et al. 2006	50	36	14	0.48	0.34	0.36	4	0.65	0.78
Passos et al. 2003	29	22	7	0.58	0.39	0.33	4	0.50	0.68
Pedro 1992	18	11	7	0.64	0.48	0.29	4	0.42	0.60
Silveira 2006	12	6	6	0.75	0.18	0.20	3	0.53	0.59

**Figure 1:**
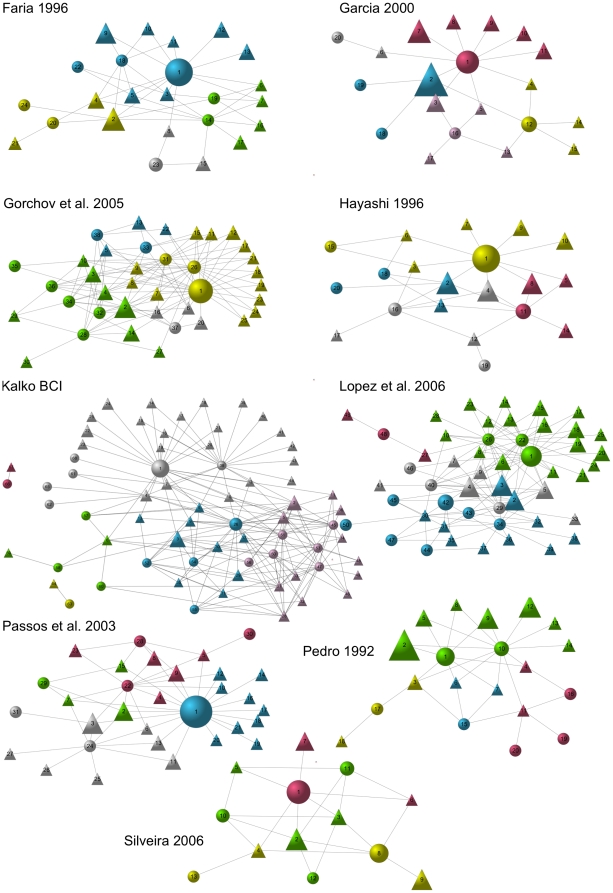
Guilds and functional roles in the networks. The studied networks have a modular structure, with primary frugivores positioned in the center of most modules, thus playing important functional roles in each guild. In those graphs, vertices represent species (circles  =  bats, triangles  =  plants), and species with more links or which are more central were represented closer to the center of the graph. The size of each vertex is proportional to how central it is in the network (betweenness centrality), i.e. how important its functional role is. Links represent interactions of frugivory and seed dispersal (lines). Colors represent modules found in our analysis. Species names follow the same numbers used in Appendix S1.

Modularity was low in the bat-fruit networks (*M* = 0.32±0.07, all P<0.01) with an average of 4±1 modules in each network (varying from 3 to 6). Is most cases, species of the three main genera of frugivorous phyllostomids were found together in the same module with the plant genera assumed to be their preferred (*Artibeus* = 0.67, G = 7.94, P = 0.004; *Carollia* = 0.78, G = 16.81, P<0.001; *Sturnira* = 0.67, G = 7.94, P = 0.004), although some plant genera were dispersed by more than one bat genus (Fig. 1).

**Figure 2:**
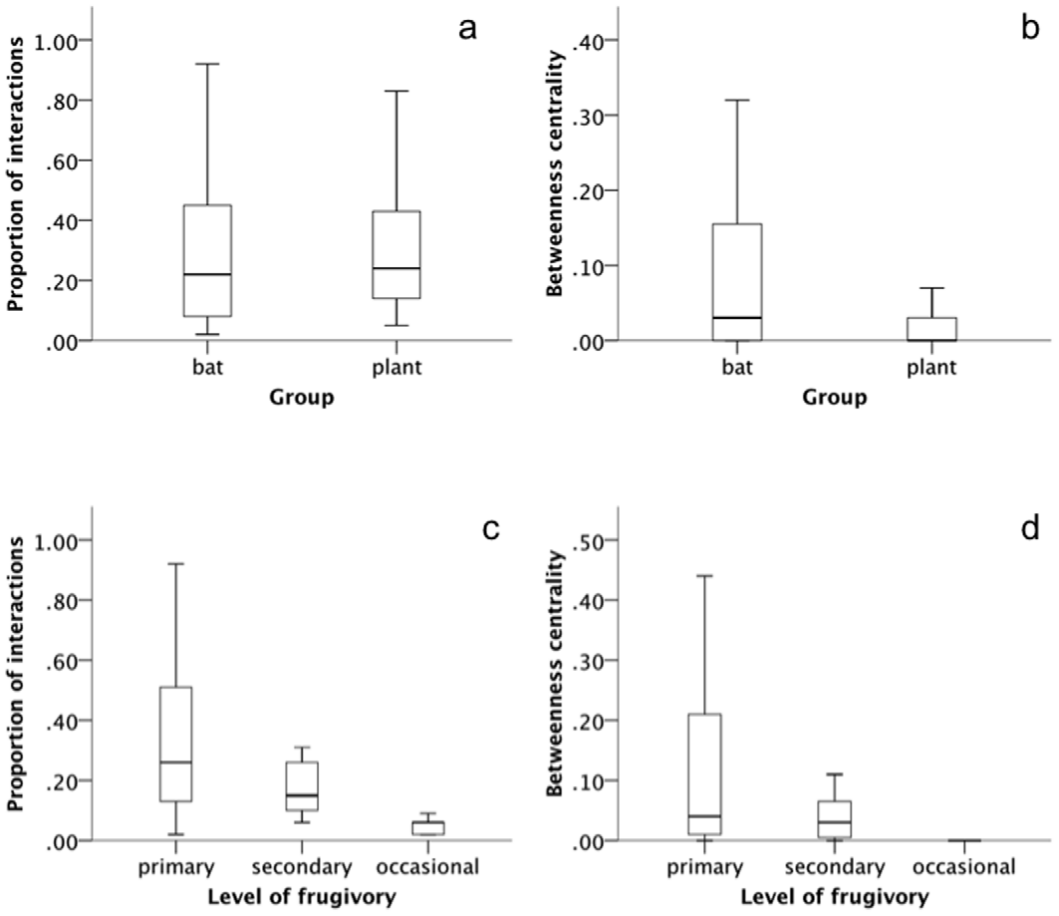
Differences among species in network properties. Interesting differences were found regarding the functional roles of different species. (a) Bats and plants interacted with a similar proportion of partners in the networks (similar proportion of interactions), whereas (b) bats occupied more central positions (higher betweenness centrality). Bat species considered as primary frugivores (c) interacted with a higher proportion of plants and (d) occupied more central positions than bat species considered as secondary or opportunistic frugivores. The main horizontal line shows the median, boxes represent quartiles, and whiskers depict 95% intervals.

Each species, bat or plant, interacted on average with about one-third of all partners available in each network. The proportion of interactions was similar between bats (*k_r_* = 0.29±0.24) and plants (*k_r_* = 0.28±0.18) (df = 94, t = 0.29, P = 0.77). Betweenness centrality was highly variable among species in each network. On average bats (*bc* = 0.10±0.15) had higher values than plants (*bc* = 0.03±0.05) (df = 94, t = 4.48, P<0.001) (Fig. 2). Primary frugivores showed higher values than secondary and occasional frugivores, both for proportion of interactions (N = 87, df = 2, K = 16.76, P<0.001) and for betweenness centrality (N = 87, df = 2, K = 9.91, P = 0.007) (Fig. 2).

**Figure 3:**
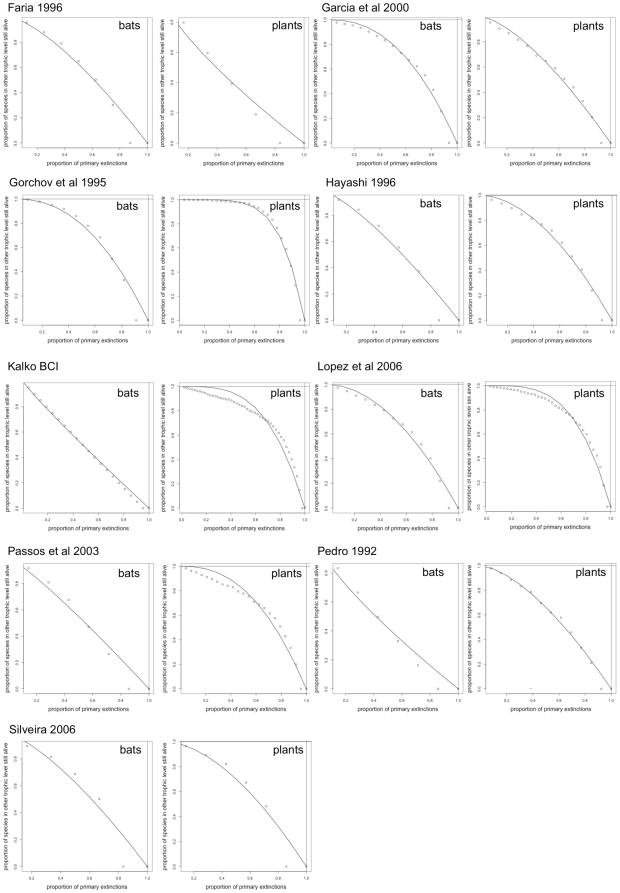
Robustness to cumulative species removal. The simulations of cumulative removals of species showed that bat-fruit networks are very robust both to removals of bats and plants, as extinction curves declined slowly on average.

**Figure 4:**
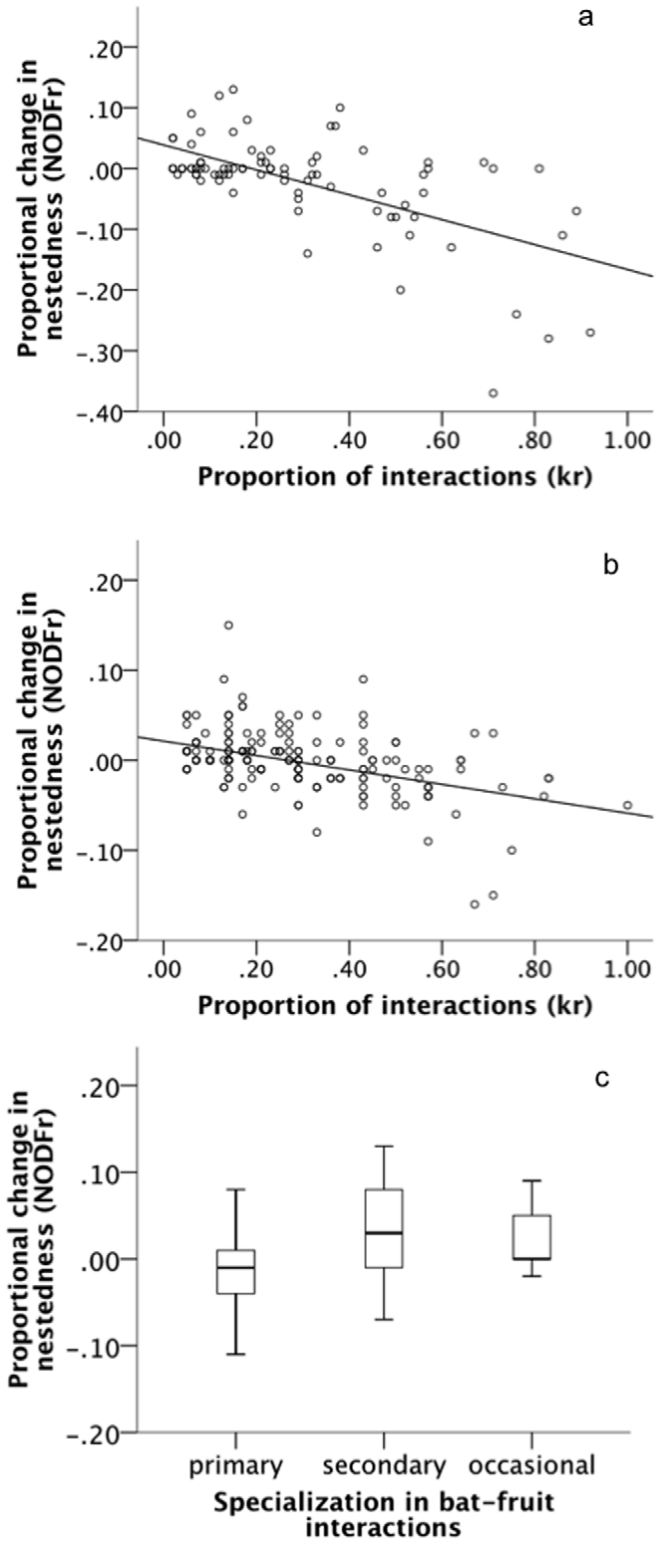
Changes caused by the removal of single species. With simulations of single-species removals, we observed that species of both (a) bats and (b) plants that interact with a higher proportion of mutualistic partners are more important for maintaining the whole network structure, as their removal causes larger decreases in nestedness. Furthermore, (c) the removal of bat species considered as primary frugivores caused also larger decreases in nestedness in the whole network.

The robustness of bat-fruit networks to cumulative extinctions was relatively high, both for bats (*R* = 0.55±0.10, range 0.41–0.69) and plants (*R* = 0.68±0.09, range 0.58–0.84) (Fig. 3). There was also a high robustness to the removal of single species. Proportional change in nestedness (*NODF_r_*) varied from 0 to 3.7%, and was lower than 1% in most cases. Furthermore, there were hardly any secondary losses (*SL_r_* = 0.0/0.00: median/quartiles, varying from 0 to 3.7%, most cases = 0). Removal of species which interacted with a higher proportion of available partners caused larger changes in nestedness in both bats (N = 87, r = −0.46, P<0.001) and plants (N = 198, r = −0.44, P<0.001) (Fig. 4). The removal of primary frugivores caused larger decreases in nestedness than the removal of secondary or occasional frugivores (N = 87, df = 2, K = 6.87, P = 0.03) (Fig. 4).

## Discussion

In our study we add an important piece to the puzzle of seed dispersal networks by describing the structure and robustness of bat-fruit networks, and showing how they are influenced by dietary specialization at both the network and species level. Bat-fruit networks showed to be robust systems with low complementary specialization, but also a modular structure, in which primary frugivores have the most important functional roles.

The first point to examine is what bat-fruit networks have in common with other networks of facultative mutualism. The pervasive nested topology observed in bat-fruit networks, as well as in many other mutualistic systems, suggests that similar processes may be structuring very different facultative mutualisms, ranging from pollination [44] to marine cleaning symbiosis [45]. Nestedness is assumed to increase resilience and biodiversity [28], since species with few interactions tend to be more fragile than species with many interactions, which form a core of highly-connected and resistant species. The cores of the studied bat-fruit networks were composed by the three main frugivorous bat genera (*Artibeus*, *Carollia* and *Sturnira*) and their five main food-plant genera (*Cecropia*, *Ficus*, *Piper*, *Solanum* and *Vismia*). Some authors have stated that a nested topology may also emerge from random networks [27]. However, this is still a controversy among network ecologists. The main reason for finding a significantly nested structure in random matrices was a conceptual error in the original nestedness formula, which was derived from the *T* metric [34]. This flaw was shown recently and a new nestedness metric has been proposed: *NODF*, which is much better tuned with the original concept of nestedness and fits better to studies on mutualism [26]. This new metric, which we used in our analysis, was not evaluated by critics of nestedness analysis, and contrary to *T*, it gives consistently low values for random matrices.

The low level of complementary specialization observed in bat-fruit networks was similar to values from bird-fruit networks (median close to 0.30) [46]. Therefore, in contrast to pollination networks, low complementary specialization does indeed seem to be a common property of seed dispersal networks. It is assumed that seed dispersal is a more diffuse interaction compared to pollination, because it is more difficult for plants to develop mechanisms that restrict access to fruits. Furthermore, to be a legitimate seed disperser, an animal has just to avoid killing the seeds and then transport them away from the mother-plant [47]. However, to be a legitimate pollinator a much finer match is required, ultimately aiming at carrying pollen from flowers of one individual to other individual plant of the same species [48]. Because specialization depends not only on the type of interaction, but also on the groups of organisms, it will be interesting in the future to study bat-flower networks in order to test if complementary specialization is higher than in bat-fruit networks.

It is interesting to note that the ecological and network concepts of specialization used in our study revealed contrasting relationships. On the one hand, according to the ecological concept of dietary specialization used here, a phyllostomid bat species that has a high level of frugivory such as *Sturnira lilium* [49], and so depends on fruits for living, may be considered as a specialist, compared to other phyllostomids viewed as generalists for feeding equally on many kinds of food (e.g. fruits, nectar, insects), such as *Phyllostomus hastatus* [50]. On the other hand, those species that are ecologically more specialized turned out to be very generalistic according to the network concept, as they interacted on many fruit species within their networks. There are differences even between species of the same genus, as for instance all *Carollia* bats are primary frugivores, but *C. perspicillata* feeds on a much larger variety of plants than *C. castanea*, at least in Barro Colorado, Panama. Therefore, we have to be careful when interpreting specialization in a network context, and we need to state clearly which ecological concept is being operationalized with which network concept. Many species identified as generalists in the network sense are probably in fact specialists according to a broad ecological concept such as dietary specialization. In this study, ecologically specialized frugivores (i.e. primary frugivores) were shown to be more important for maintaining the whole network structure. In this way, compared with other studies we have gone one step further in the assessment of the functional role of different species in mutualistic networks, because we have directly linked network importance to functional role.

Intermediate nestedness, low complementary specialization and low modularity seem to lead to a cohesive structure with a balance between redundancy within modules and complementarity among modules, because some key bat genera are responsible mainly for dispersing their preferred plant genera, and so each network is composed of modules with a phylogenetic signal. It is interesting to notice that the genus-genus associations uncovered in population studies of bats and plants [18] seem to influence the structure of modules within bat-fruit networks. The relationships between *Artibeus*-*Ficus*, *Carollia*-*Piper*, and *Sturnira*-*Solanum* were consistent among different local networks despite some geographical variations. In fact, those geographic variations can be explained by differences in the local availability of plant species upon which frugivorous bats feed, which is caused mainly by differences in the geographic distribution of bats and plants that interact with each other. This pattern reinforces the assumed background of a coevolutionary history between those bat and plant genera [51]. Among ecologically similar species, such as those of the genera *Carollia* and *Sturnira*, it is very likely that a couple of factors, in particular fruit secondary metabolites, play major roles [52], [53] in permitting resource partitioning, and ultimately their coexistence [54].

Specialization and redundancy might explain the high robustness observed in bat-fruit networks. High robustness to random removal of nodes (‘error’ in the network jargon) is a common feature of many complex networks [55], including mutualistic networks [56]. It is typical of networks with a scale-free or broad-scale topology, where only a few species have a disproportionally high number of interactions, and most species have few interactions [55]. In the case of bat-fruit systems, this tolerance is probably enhanced by the high redundancy. It seems that within each module, bats of the same genus play redundant roles in the dispersal of plant species of their preferred genera. In turn, modules are complementary to each other, as species in each module are responsible for a particular part of the whole dispersal service. Finally, despite those genus-genus associations, many plant genera are dispersed also by other bat genera and not only their main partners. The result is a robust system, in which there are back-ups both within and outside each module, ensuring that most plant species continue to be serviced even in the absence of their main mutualists. This finding is of great relevance to conservation, as bats are predominantly involved in seed dispersal services at pioneer stages, therefore being the main group responsible for forest regeneration [10], [11].

In conclusion, dietary specialization (here assessed as level of frugivory) seems to be an important structuring factor in bat-fruit networks. It would be interesting for future studies to go one step further and study how physiological differences among bat species (e.g. the ability to cope with particular secondary metabolites) may explain niche segregation at network level. After more studies are conducted, we may be able to use network properties to help define conservation priorities and even restore degraded areas in a more efficient way. For example, plant species pointed out as hubs in seed dispersal networks can be good candidates for reforestation programs, as they are likely to attract more disperser species and accelerate regeneration. And species identified as connectors may be also important, as they will help to increase the system's cohesiveness and, ultimately, robustness.

## Supporting Information

Appendix S1 Datasets on bat-fruit interactions used in our analysis.
(PDF)

Appendix S2 Matrices analyzed in this study.
(PDF)
