# Supplementary material for: The Missing Part of Seed Dispersal Networks: Structure and Robustness of Bat-Fruit Interactions
Source: PLoS One. 2011 Feb 28;6(2):e17395. doi: 10.1371/journal.pone.0017395 (PMC3046224; doi:10.1371/journal.pone.0017395)
Supplement: Appendix S1 — Datasets on bat-fruit interactions used in our analysis. (PDF) [file pone.0017395.s001.pdf]

Supplementary Information

Appendix 1: Datasets on bat-fruit interactions used in our analysis.

1. Faria DM (1996) Uso de recursos alimentares por morcegos filostomídeos fitófagos na Reserva de Santa Genebra, Campinas, São Paulo. MSc Dissertation. Campinas: Universidade Estadual de Campinas.

2. Garcia QS, Rezende JL, Aguiar LMS (2000) Seed dispersal by bats in a disturbed area of southeastern Brazil. Revista de Biologia Tropical 48: 125-128.

3. Giannini NP, Kalko EKV (2004) Trophic structure in a large assemblage of phyllostomid bats in Panama. Oikos 105: 209-220. Obs: this dataset was complemented with further data collected thereafter, and is referred to as “Kalko BCI” in our paper.

4. Gorchov DL, Cornejo F, Ascorra CF, Jaramillo M (1995) Dietary overlap between frugivorous birds and bats in the Peruvian Amazon. Oikos 74: 235-250.

5. Hayashi MM (1996) Morcegos frugívoros em duas áreas alteradas da fazenda Lageado, Botucatu, Estado de São Paulo. MSc Dissertation. Botucatu: Universidade Estadual Paulista.

6. Lopez JE, Vaughan C (2004) Observations on the role of frugivorous bats as seed dispersers in costa rican secondary humid forests. Acta Chiropterologica 6: 111-119.

7. Passos FC, Silva WR, Pedro WA, Bonin MR (2003) Frugivoria em morcegos (Mammalia, Chiroptera) no Parque Estadual Intervales, sudeste do Brasil. Revista Brasileira de Zoologia 20: 511-517.

8. Pedro WA (1992) Estrutura de uma taxocenose de morcegos da Reserva do Panga (Uberlândia, MG), com ênfase nas relações tróficas em Phyllostomidae (Mammalia: Chiroptera). MSc Dissertation. Campinas: Universidade Estadual de Campinas.

9. Silveira M (2006) Dispersão de sementes por morcegos frugívoros em uma área em processo de restauração vegetal na RPPN Parque Florestal São Marcelo Mogi-Guaçu-SP. BSc Monograph. Rio Claro: Universidade Estadual de São Paulo. 43 p.
