# Supplementary material for: The Missing Part of Seed Dispersal Networks: Structure and Robustness of Bat-Fruit Interactions
Source: PLoS One. 2011 Feb 28;6(2):e17395. doi: 10.1371/journal.pone.0017395 (PMC3046224; doi:10.1371/journal.pone.0017395)
Supplement: Appendix S2 — Matrices analyzed in this study. (PDF) [file pone.0017395.s002.pdf]

Appendix 2: Matrices analyzed in this study.

Faria 1996

		9 Solanum_granulosoleprosum	10 Piper_sp2	11 Ficus_luschnathiana	12 Polpa_sp1	13 Ficus_enormis	14 Polpa_sp2	15 Ficus_sp1	16 Ficus_insipida	17 Cecropia_hololeuca	18 Cecropia_pachystachya	19 Ficus_sp2	20 Piper_amalago	21 Piper_arboreum	22 Maclura_tinctoria	23 Muntingia_calabura	24 Solanum_aequale
1	Artibeus_lituratus	17	1	9	4	4	9	5	5	5	3	4	0	1	0	0	0
2	Sturnira_lilium	22	2	0	1	1	0	1	0	0	0	0	1	1	0	1	2
3	Carollia_perspicillata	7	12	0	1	0	0	0	0	0	0	0	2	1	0	0	0
4	Platyrrhinus_lineatus	1	0	1	1	3	0	1	2	0	3	0	0	0	0	0	0
5	Pygoderma_bilabiatum	2	0	0	0	1	0	0	0	0	0	0	0	0	2	0	0
6	Chiroderma_doriae	0	0	0	3	0	0	1	0	0	0	0	0	0	0	0	0
7	Glossophaga_soricina	0	0	0	0	0	0	0	0	1	0	0	0	0	0	1	0
8	Phyllostomus_discolor	2	0	0	0	0	0	0	0	0	0	0	0	0	0	0	0

Garcia et al. 2000

		7 Cecropia_glaziovi	8 Solanaceae_sp4	9 Piper_sp	10 Psidium_sp1	11 Psidium_sp2	12 Piper_aduncum	13 Carica_papaya	14 Ficus_sp1	15 Ficus_sp2	16 Ficus_sp3	17 Miconia_sp	18 Solanaceae_sp1	19 Solanaceae_sp2	20 Solanaceae_sp3
1	Carollia_perspicillata	4	5	3	0	0	0	0	0	0	0	0	0	1	1
2	Artibeus_lituratus	2	0	1	2	1	1	1	1	1	1	1	0	0	0
3	Sturnira_lilium	0	2	0	1	0	1	0	0	0	0	0	1	0	0
4	Vampyressa_pussila	0	0	0	0	2	0	0	0	0	0	0	0	0	0
5	Glossophaga_soricina	1	0	0	0	0	0	0	0	0	0	0	0	0	0
6	Plathyrrhinus_recifinus	1	0	0	0	0	0	0	0	0	0	0	0	0	0

Gorchov et al. 1995

		12 Vismia_angusta	13 Piper_aduncum	14 Cecropia_ficifolia	15 Cecropia_distachya	16 Vismia_macrophylla	17 Cecropia_membranacea	18 Piper_arboreum	19 Solanum_lanceolatum	20 Ficus_sp2	21 Araceae1	22 Piper_sp2	23 Guttiferae1	24 Cecropia_sciadophylla	25 Amaranthaceae1	26 Anthurium_sp1	27 Piperaceae1	28 Pothomorphe_peltata	29 Passifloraceae1	30 Ficus_sp3	31 Ficus_sp1	32 Gurania_acuminata	33 Piper_sp1.	34 Solanum_jamaicense	35 Philodendron_sp	36 Piperaceae2	37 unknown1	38 Vismia_sp
1	Carollia_perspicillata	152	127	29	4	35	2	17	13	3	4	9	1	2	9	3	2	1	4	0	1	0	1	1	1	2	0	1
2	Carollia_brevicauda	80	51	17	2	11	1	8	4	2	9	5	0	0	1	2	4	6	2	0	0	1	2	1	2	1	0	1
3	Phyllostomus_hastatus	1	3	5	49	0	32	0	0	0	0	0	0	10	0	1	0	0	0	1	0	3	0	0	0	0	3	0
4	Carollia_castanea	9	18	1	0	3	1	6	1	2	1	1	0	0	1	1	1	0	0	0	0	0	1	0	0	0	0	0
5	Artibeus_lituratus	2	1	18	6	1	5	0	0	2	0	0	0	0	0	0	0	0	0	0	0	0	0	0	0	0	0	0
6	Rhinophylla_pumilio	13	0	2	2	3	0	0	0	0	1	0	12	0	1	0	0	0	0	0	0	0	0	0	0	0	0	0
7	Artibeus_jamaicensis	3	1	12	2	0	0	1	0	4	0	0	0	1	0	1	0	0	0	1	0	0	0	0	0	0	0	0
8	Artibeus_obscurus	0	0	19	0	0	0	0	0	2	0	0	0	0	0	0	0	0	0	0	0	0	0	0	0	0	0	0
9	Uroderma_bilobatum	1	1	1	0	0	0	1	3	4	0	0	0	0	0	0	0	0	0	2	0	0	0	0	0	0	0	0
10	Artibeus_gnomus	3	0	3	0	1	0	0	0	0	0	0	0	0	0	2	0	0	0	0	3	0	0	0	0	0	0	0
11	Sturnira_lilium	0	1	4	1	0	0	0	2	0	0	0	1	0	0	0	0	0	0	0	0	0	0	2	0	0	0	0

Hayashi 1996

		8 Solanum_erianthum	9 Piper_crassinervium	10 Ficus_retusa	11 Cecropia_sp	12 unidentified1	13 Ficus_benjamina	14 Piper_gaudichaudianum	15 Piper_amalago	16 Eriobotrya_japonica	17 Mangifera_indica	18 Piper_sp	19 Muntingia_calabura	20 Solanum_paniculatum
1	Sturnira_lilium	39	9	0	1	3	0	1	3	1	0	2	0	1
2	Artibeus_lituratus	2	0	2	7	3	3	0	0	1	2	0	0	0
3	Carollia_perspicillata	0	12	0	0	0	0	2	0	0	0	0	0	0
4	Platyrrhinus_lineatus	2	1	8	1	0	1	0	0	0	0	0	1	0
5	Glossophaga_soricina	1	0	0	0	0	1	1	0	0	0	0	0	0
6	Chiroderma_doriae	0	0	2	0	0	0	0	0	0	0	0	0	0
7	Pygoderma_bilabiatum	1	0	0	0	0	0	0	0	0	0	0	0	0

		22	23	24	25	26	27	28	29	30	31	32	33	34	35	36	37	38	39	40	41	42	43	44	45	46	47	48	49	50	51	52	53	54	55	56
		Ficus_insipida	Ficus_yoponensis	Ficus_sp	Ficus_trigonata	Spondias_mombin	Ficus_obtusifolia	Cecropia_spp.	Piper_sp.	Anacardium_excelsum	Ficus_citrifolia	Ficus_costaricana	Spondias_radlkoferi	Ficus_bullenei	Ficus_popenoei	Quararibea_asterolepis	Dipteryx_panamensis	Ficus_dugandii	Ficus_maxima	Ficus_nymphaeifolia	Poulsenia_armata	Trichilia_sp	Brosimum_alicastrum	Calophyllum_longifolium	Ficus_pertusa	Ficus_tonduzii	Havetiopsis_flexilis	Solanum_sp.	Philodendron_sp	Piper_arboreum	Piper_cordulatum	Piper_grande	Piper_marginatum	Piper_reticulatum	Eugenia_spp.	Ficus_paraensis
1	Carollia_perspicillata	1	0	1	0	1	1	1	1	1	0	0	1	0	0	1	1	0	0	0	0	1	1	0	0	0	1	1	1	1	1	1	1	1	1	0
2	Artibeus_jamaicensis	1	1	1	1	1	1	1	1	1	1	1	1	1	1	1	1	0	1	1	1	1	1	1	0	1	0	0	0	0	0	0	0	0	0	0
3	Carollia_castanea	1	0	0	0	1	0	0	1	1	0	0	0	0	0	1	0	0	0	0	0	1	0	0	0	0	1	1	1	1	1	1	1	1	0	0
4	Artibeus_lituratus	1	1	1	1	1	1	0	1	0	0	1	1	0	1	1	1	1	0	1	0	0	0	1	0	0	0	0	0	0	0	0	0	0	0	0
5	Uroderma_bilobatum	1	1	0	1	0	1	0	0	0	1	1	0	1	1	1	1	1	1	1	0	0	0	0	1	0	0	0	0	0	0	0	0	0	0	1
6	Vampyressa_nymphaea	1	1	1	1	0	1	1	0	0	1	0	0	0	1	0	0	1	1	1	0	0	0	0	0	0	0	0	0	0	0	0	0	0	0	0
7	Vampyrodes_caraccioli	1	1	0	1	1	1	0	1	0	0	0	1	0	0	0	0	0	1	0	1	0	0	1	1	0	0	0	0	0	0	0	0	0	0	0
8	Artibeus_watsoni	0	1	1	1	0	0	0	0	1	1	1	1	1	0	0	0	0	0	0	1	0	1	0	0	0	0	0	0	0	0	0	0	0	0	0
9	Chiroderma_villosum	1	1	1	1	0	1	0	0	0	1	0	0	0	0	0	0	1	0	0	0	1	0	0	1	1	0	0	0	0	0	0	0	0	0	0
10	Artibeus_phaeotis	1	1	1	0	0	0	0	0	1	0	1	1	1	0	0	0	0	0	0	1	0	0	0	0	0	0	0	0	0	0	0	0	0	0	0
11	Vampyressa_pusilla	0	1	1	1	0	0	0	0	0	1	1	0	1	1	0	0	0	0	0	0	0	0	0	0	0	0	0	0	0	0	0	0	0	0	0
12	Platyrrhinus_helleri	1	1	1	1	0	1	1	0	0	0	0	0	0	0	0	0	0	0	0	0	0	0	0	0	0	0	0	0	0	0	0	0	0	0	0
13	Phyllostomus_discolor	1	0	0	0	1	0	1	0	1	0	0	0	0	0	0	0	0	0	0	0	0	0	0	0	0	0	0	0	0	0	0	0	0	0	0
14	Glossophaga_soricina_	0	0	1	0	0	0	1	0	0	0	0	0	0	0	0	0	0	0	0	0	0	0	0	0	0	0	0	0	0	0	0	0	0	0	0
15	Phyllostomus_hastatus	0	1	0	1	0	0	1	0	0	0	0	0	0	0	0	0	0	0	0	0	0	0	0	0	0	0	0	0	0	0	0	0	0	0	0
16	Lampronycteris_brachyotis	0	0	0	0	1	0	0	1	0	0	0	0	0	0	0	0	0	0	0	0	0	0	0	0	0	0	0	0	0	0	0	0	0	0	0
17	Carollia_brevicauda	0	0	0	0	1	0	0	0	0	0	0	0	0	0	0	0	0	0	0	0	0	0	0	0	0	0	0	0	0	0	0	0	0	0	0
18	Centurio_senex	0	0	0	0	0	0	0	0	0	0	0	0	0	0	0	0	0	0	0	0	0	0	0	0	0	0	0	0	0	0	0	0	0	0	0
19	Micronycteris_hirsuta	0	0	0	0	1	0	0	0	0	0	0	0	0	0	0	0	0	0	0	0	0	0	0	0	0	0	0	0	0	0	0	0	0	0	0
20	Phylloderma_stenops	0	0	0	0	0	0	0	0	0	0	0	0	0	0	0	0	0	0	0	0	0	0	0	0	0	0	0	0	0	0	0	0	0	0	0
21	Trinycteris_nicefori	0	0	0	0	0	0	0	1	0	0	0	0	0	0	0	0	0	0	0	0	0	0	0	0	0	0	0	0	0	0	0	0	0	0	0

Lopez & Vaughn 2004

		15 Piper_sancti-felicis	16 Piper_auritum	17 Piper_multiplinervium	18 Vismia_panamensis	19 Cecropia_obtusifolia	20 Solanum_rugosum	21 Ficus_cahuitensis	22 Piper_glabratum	23 Piper_colonense	24 Piper_friedrichsthalii	25 Philodendron_sp	26 Ficus_insipida	27 Markea_neuratha	28 Piper_trigonum	29 Piper_sp3	30 Cecropia_insignis	31 Piper_augustum	32 Senna_fruticosa	33 Piper_reticulatum	34 Piper_sp2	35 Solanum_arboreum	36 Ficus_popenoei	37 Ficus_nymphaeifolia	38 Piper_aduncum	39 Piper_sp1	40 Hernandia_didymantha	41 Anthurium_sp	42 Passiflora_sp	43 Pothomorphe_peltata	44 Annona_sp.	45 Ficus_pertusa	46 Piper_hispidum	47 Piper_sp4	48 Piper_sp5	49 Clarisia_mexicana
1	Carollia_castanea	69	2	64	4	0	7	0	29	12	17	2	0	0	9	5	0	1	1	3	3	2	0	0	2	4	0	0	0	1	0	0	0	0	1	0
2	Carollia_sowelli	43	58	21	25	0	19	0	7	13	2	14	0	5	5	2	0	5	1	0	3	2	0	0	2	0	0	3	0	1	0	0	0	1	0	0
3	Carollia_perspicillata	17	29	33	21	0	15	0	6	2	9	3	0	2	0	1	0	1	5	3	0	2	0	0	1	0	0	0	1	0	0	0	1	0	0	0
4	Artibeus_jamaicensis	1	1	0	5	23	1	32	0	0	0	0	16	0	0	0	0	0	0	0	0	0	1	4	0	0	3	0	0	0	0	0	0	0	0	1
5	Dermanura_sp	14	19	1	2	16	1	1	5	6	0	1	0	1	0	0	0	0	0	0	0	0	2	0	0	0	0	0	0	0	0	1	0	0	0	0
6	Glossophaga_commissarisi	2	14	0	25	1	1	0	2	1	1	1	0	5	0	0	8	0	0	0	0	0	0	0	0	0	0	0	0	0	0	0	0	0	0	0
7	Vampyressa_nymphaea	1	0	0	0	5	4	3	0	0	0	0	0	0	0	0	0	0	0	0	0	0	3	0	0	0	0	0	0	0	0	0	0	0	0	0
8	Vampyrops_helleri	0	0	0	0	10	0	3	0	0	0	0	1	0	0	0	0	0	0	0	0	0	0	0	0	0	0	0	0	0	0	0	0	0	0	0
9	Artibeus_lituratus	0	1	0	4	4	0	2	0	0	0	0	1	0	0	0	0	0	0	0	0	0	0	1	0	0	0	0	0	0	0	0	0	0	0	0
10	Chiroderma_villosum	0	0	0	0	1	0	8	0	0	0	0	0	0	0	0	0	0	0	0	0	0	0	0	0	0	1	0	0	0	0	0	0	0	0	0
11	Uroderma_bilobatum	1	0	0	0	1	3	1	0	0	0	0	0	0	0	0	0	0	0	0	0	0	0	0	0	0	0	0	0	0	0	0	0	0	0	0
12	Hylonycteris_underwoodi	0	0	0	1	0	0	0	0	0	0	0	0	3	0	0	0	0	0	0	0	0	0	0	0	0	0	0	0	0	0	0	0	0	0	0
13	Phylloderma_stenops	0	0	0	0	0	0	0	0	0	0	0	0	0	0	0	0	0	0	0	0	0	0	0	0	0	0	0	1	0	2	0	0	0	0	0
14	Vampyressa_pusilla	0	0	0	0	1	0	0	0	0	0	0	0	0	0	0	0	0	0	0	0	0	0	0	0	0	0	0	0	0	0	0	0	0	0	0

Passos et al. 2003

		8 Solanum_sanctae-katharinae	9 Vismia_sp	10 Solanum_swartzianum	11 Ficus_sp	12 Piper_dilatatum	13 Polpa_sem_semente	14 Cecropia_glaziovi	15 Ficus_luschnathiana	16 Piper_aduncum	17 Vassobia_breviflora	18 Solanum_subsylvestris	19 Cecropia_pachystachya	20 Familias_indeterminadas	21 Piper_sp	22 Solanum_megalochiton	23 Solanum_rufescens	24 Solanum_scuticum	25 Solanum_sp	26 Philodendron_apendiculatum	27 Aureliana_sp	28 Rubus_brasiliensis	29 Solanum_paranaense	30 Solanum_cinnamomeum	31 Solanum_variabile
1	Sturnira_lilium	15	8	11	1	4	2	1	1	0	1	3	2	0	1	3	1	3	3	0	1	1	0	1	1
2	Artibeus_fimbriatus	1	4	1	5	1	1	3	3	0	4	0	1	2	0	0	0	0	0	0	0	0	0	0	0
3	Carollia_perspicillata	1	2	0	0	2	2	0	0	5	0	1	0	0	2	0	0	0	0	2	0	0	1	0	0
4	Artibeus_lituratus	0	0	0	3	0	0	1	1	0	0	0	0	1	0	0	1	0	0	0	0	0	0	0	0
5	Sturnira_tildae	3	0	0	0	0	1	0	0	0	0	0	0	0	0	0	1	0	0	0	0	0	0	0	0
6	Artibeus_jamaicensis	0	0	0	1	0	0	0	0	0	0	0	0	0	0	0	0	0	0	0	0	0	0	0	0
7	Pygoderma_bilabiatum	0	1	0	0	0	0	0	0	0	0	0	0	0	0	0	0	0	0	0	0	0	0	0	0

Pedro 1992

		8 Piper_amalago	9 Cecropia_pachystachya	10 unidentified1	11 Piper_gaudichaudianum	12 Ficus_sp	13 Semente_não_identificada	14 Piper_arboreum	15 Solanum_sp3	16 Piper_sp	17 Solanum_inaequale	18 Copaifera_langsdorffii	19 Solanum_paniculatum	20 Miconia_albicans
1	Carollia_perspicilatta	24	1	7	11	0	1	1	0	2	1	0	0	0
2	Sturnira_lilium	5	1	6	1	1	4	2	4	1	2	2	2	0
3	Vampyrops_lineatus	0	14	0	0	4	3	1	0	0	0	0	0	0
4	Artibeus_planirostris	0	0	2	0	1	0	0	0	0	0	0	0	0
5	Glossophaga_soricina	0	1	0	0	0	0	0	0	0	0	0	0	1
6	Artibeus_lituratus	0	0	0	0	1	0	0	0	0	0	0	0	0
7	Chiroderma_doriae	0	0	0	0	1	0	0	0	0	0	0	0	0

Silveira 2006

		7 Cecropia_pachystachya	8 Solanum_sp	9 Fícus_sp	10 Ficus_guarantica	11 Piper_aduncum	12 Famílias_indeterminadas	13 Photomorpha_umbellata
1	Artibeus_lituratus	25	36	2	4	2	2	0
2	Carollia_perspicillata	2	25	1	0	11	0	3
3	Platyrrhinus_lineatus	5	19	1	4	0	0	0
4	Sturnira_lilium	3	30	0	1	2	0	0
5	Glossophaga_soricina	2	1	0	0	0	0	0
6	Vampyressa_cf._pusilla	0	0	1	0	0	0	0
